# Ossification of the Pulp of a Tooth

**Published:** 1845-03

**Authors:** 


*Ossification of the Pulp of a Tooth.*—The crowns of the teeth of some presons, as is well known to most dentists, from the peculiar manner in which they come together, are more liable to be worn off, than those of others. Occurrences of this sort are not unfrequent, and but for a singular and beautiful provision of nature, consisting in the ossification of their pulps, would expose their cavities and be attended with the most painful consequences. But the ossification of the pulps of the teeth, under any other circumstances, is of rare occurrence, and especially in young persons. We, however, have several teeth in our collection of morbid dental specimens, in which this has taken place, but one of the most remarkable examples of the sort, we recollect to have ever met with, was presented us by Dr. Cassell, of this city. The tooth in which it had taken place, is an upper central incisor, and apparently from the mouth of a person not over sixteen or eighteen years of age. The pulp is completely ossified and isolated, except at a single point from the surrounding walls of the tooth.—*Balt. Ed.*

